# Spectral reconstruction of signals from periodic nonuniform subsampling based on a Nyquist folding scheme

**DOI:** 10.1186/s13634-017-0458-z

**Published:** 2017-02-23

**Authors:** Kaili Jiang, Jun Zhu, Bin Tang

**Affiliations:** School of Electronic Engineering, University of Electronic Science and Technology of China, Qingshuihe Campus, No.2006, Xiyuan Ave, West Hi-Tech Zone, Chengdu, Sichuan China

**Keywords:** Nyquist folding receiver, Periodic nonuniform subsampling, Spectral reconstruction

## Abstract

Periodic nonuniform sampling occurs in many applications, and the Nyquist folding receiver (NYFR) is an efficient, low complexity, and broadband spectrum sensing architecture. In this paper, we first derive that the radio frequency (RF) sample clock function of NYFR is periodic nonuniform. Then, the classical results of periodic nonuniform sampling are applied to NYFR. We extend the spectral reconstruction algorithm of time series decomposed model to the subsampling case by using the spectrum characteristics of NYFR. The subsampling case is common for broadband spectrum surveillance. Finally, we take example for a LFM signal under large bandwidth to verify the proposed algorithm and compare the spectral reconstruction algorithm with orthogonal matching pursuit (OMP) algorithm.

## Introduction

Under the condition of modern information warfare, reconnaissance receiver faces the gradually complex electromagnetic environment; accompanied by diversification of electromagnetic radiation sources and coexistence of jamming and anti-jamming. The features of received signals are wide time-frequency-space domain, waveform complexity, and large dynamic range. So to speak, the problem is receiving and dealing with the wideband signals. In recent years, with the rapid development of radar technology, the range of the frequency spectrum is from 5 MHz to 95 GHz and enlarges gradually [1]. The existing reconnaissance receiver cannot match the coverage of radar because of the limited sampling rate and precision of analog-to-digital converter (ADC) [2]. Therefore, how to solve this problem becomes a focus.

Reconnaissance receiver as a channelized receiver [3, 4], in general, is based on the Nyquist theorem for design of the data acquisition of wideband signals [5]. And Nyquist rate is only a necessary but not sufficient condition for signals recovered accurately [6]. For another, nonuniform sampling exists extensively in the practical system of nonideal and compressed sensing (CS) theory as a typical example of nonuniform sampling. The research in analog-to-information (A2I) conversion is still limited in prototype and numerical simulation [7]. And there are some requirements for the sparse characteristic of the received signals based on CS [8–11].

Periodic nonuniform sampling introduces enough nonuniform to differentiate the frequency band of the received signals, whose randomness of sampling is between uniform sampling and random sampling. JENQ presents the detailed Fourier spectrum and digital spectrum of periodic nonuniformly sampled signals by a time series decomposed model [12], and its spectral reconstruction algorithm under the Nyquist theorem described in the reference [13]. Similarly, the fractional Fourier spectrum of periodic nonuniformly sampled signals and the fractional spectral reconstruction are discussed by Ran Tao [14, 15], for linear frequency modulation (LFM) signals. However, the spectral reconstruction of periodic nonuniform subsampling based on Fourier or fractional Fourier has not been reported by far.

**Fig. 1:**
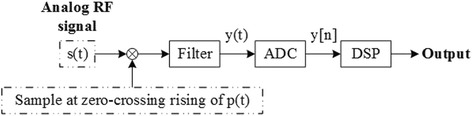
The Nyquist folding receiver architecture

Nyquist folding receiver (NYFR) [16] is a secondary sampling scheme as shown in Fig. 1. It modulates multiple Nyquist zones first by a stream of short pluses. And we show that the first radio frequency (RF) sampling of the NYFR is periodic nonuniform. Then, the modulated signals through a low-pass interpolation filter and digitized by ADC as the second sampling. The reference [17] shows the spectral reconstruction of multiple single frequency signals of NYFR by OMP. And they use restricted isometry property (RIP) constantly to determine the amount of sparsity needed for signal recovery as shown in reference [18]. Rather in the reference [19], the NYFR architecture is analyzed based on the RIP and block RIP; then, the output signal is recovered by block CS algorithm. Meanwhile, the signal detection and parameter estimation algorithms are studied in references [20, 21]. In this paper, we will give the spectral reconstruction algorithm of periodic nonuniform subsampling based on this architecture.

## Periodic nonuniform sampling

NYFR folds broadband RF inputs to a low-pass interpolation filter with a steam of short pluses. The time of the short pluses corresponds to zero-crossing rising time of the RF sample clock function, and the modulated phase of the RF sample clock function may be sinusoid frequency modulation (SFM), linear frequency modulation (LFM), etc. Then, the RF sample clock function can be assumed as

1 $$ p(t)= \sin \left(2\pi {f}_s t+\theta (t)\right) $$

where *f*_*s*_ is the average sampling frequency, and *θ*(*t*) is the phase modulation function.

The following section provides a proof that NYFR is a periodic nonuniform sampling scheme, which means the RF sample clock function is periodic nonuniform. In this paper, we assume *θ*(*t*) is a sinusoid function as an example. Note that the proof is fit for the other phase modulation function. So the RF sample clock described in Eq. (1) can be rewritten as follows:

2 $$ p(t)= \sin \left(2\pi {f}_s t+ \sin \left(2\pi {f}_{\theta} t\right)\right) $$

where *f*_*θ*_ is the frequency of the sinusoid phase modulation function.

### Periodicity

Assuming the stream of short pluses changed periodicity with *T*′, then

3 $$ \begin{array}{l} p\left( t+{T}^{\prime}\right)= \sin \left(2\pi {f}_s\left( t+{T}^{\prime}\right)+ \sin \left(2\pi {f}_{\theta}\left( t+{T}^{\prime}\right)\right)\right)\\ {}\kern6.5em = \sin \left(2\pi {f}_s t+2\pi {f}_s{T}^{\prime }+ \sin \left(2\pi {f}_{\theta} t+2\pi {f}_{\theta}{T}^{\prime}\right)\right)\end{array} $$

Following reference [16] with *f*_*s*_ ≫ *f*_*θ*_, assuming *f*_*s*_ = *Mf*_*θ*_ (*M* ∈ *Z*), it is shown that *T*′ = 1/*f*_*θ*_ and

4 $$ \begin{array}{l} p\left( t+{T}^{\prime}\right)= \sin \left(2\pi {f}_s t+2\pi {f}_s/{f}_{\theta}+ \sin \left(2\pi {f}_{\theta} t+2\pi \right)\right)\\ {}\kern6.5em = \sin \left(2\pi {f}_s t+2\pi M+ \sin \left(2\pi {f}_{\theta} t+2\pi \right)\right)\\ {}\kern4.4em = \sin \left(2\pi {f}_s t+ \sin \left(2\pi {f}_{\theta} t\right)\right)\\ {}\kern4.6em = p(t)\end{array} $$

If *f*_*s*_ ≠ *Mf*_*θ*_ (*M* ∈ *Z*), of course, the sampling period is *f*_*lcm*_ = *lcm*{*f*_*s*_, *f*_*θ*_}, that is to say the least common multiple (LCM) of {*f*_*s*_, *f*_*θ*_} when *f*_*s*_ is not multiple of *f*_*θ*_. Thus, *T*′ = 1/*f*_*lcm*_ = 1/(*l*_*s*_ *f*_*s*_) = 1/(*l*_*θ*_ *f*_*θ*_) and then the *p*(*t* + *T*′) can be expressed as

5 $$ \begin{array}{l} p\left( t+{T}^{\prime}\right)= \sin \left(2\pi {f}_s\left( t+{T}^{\prime}\right)+ \sin \left(2\pi {f}_{\theta}\left( t+{T}^{\prime}\right)\right)\right)\\ {}\kern6.5em = \sin \left(2\pi {f}_s t+2\pi {f}_s/\left({l}_s{f}_s\right)+ \sin \left(2\pi {f}_{\theta} t+2\pi {f}_{\theta}/\left({l}_{\theta}{f}_{\theta}\right)\right)\right)\\ {}\kern6.5em = \sin \left(2\pi {f}_s t+2\pi /{l}_s+ \sin \left(2\pi {f}_{\theta} t+2\pi /{l}_{\theta}\right)\right)\end{array} $$

Besides, considering an extreme case, if *f*_*s*_ and *f*_*θ*_ are co-prime, which will introduce much randomization, whose randomness of sampling is between uniform sampling and random sampling. Then, the influence of aliasing will be suppressed, and the original information of inputs will be more complete at the cost of increased algorithm complexity.

The focus of this paper is not on how to set the parameter of NYFR more suitable. However, it is given a further understanding of NYFR architecture based on periodic nonuniform sampling.

### Nonuniformity

The zero-crossing rising time *t*_*n*_ of the RF sampling clock function *p*(*t*) can be viewed as

6 $$ {\displaystyle \sum_{n=0}^N2\pi \delta \left( t-{t}_n\right)}= z c r\left\{ \sin \left(2\pi {f}_s t+ \sin \left(2\pi {f}_{\theta} t\right)\right)\right\} $$

where *zcr*{⋅} denotes sampling of the zero-crossing rising time and *N* is the number of samples. And the phase can be denoted as

7 $$ \phi (t)=2\pi {f}_s t+ \sin \left(2\pi {f}_{\theta} t\right). $$

**Fig. 2:**
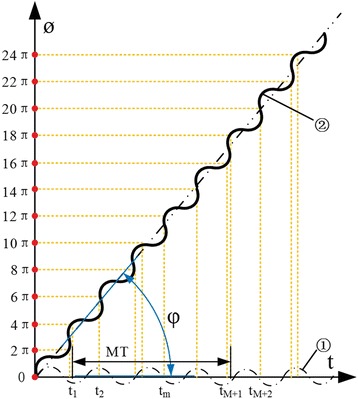
The sinusoid frequency modulation phase changes with time

Figure 2 is the sketch of sinusoid frequency modulated phase *ϕ* changes with time *t*. The sine curve② is sin(2*πf*_*θ*_ *t*) and with a slope is 2*πf*_*s*_ = tan(*φ*) ≫ 1, which means the rotation angle value is *φ* > 45°. And its projection along the time axis is nonuniform as shown in curve of ①. Note that the projection of sinusoid phase modulation function is uniform when *φ* = 0° *or* 45°.

**Fig. 3:**
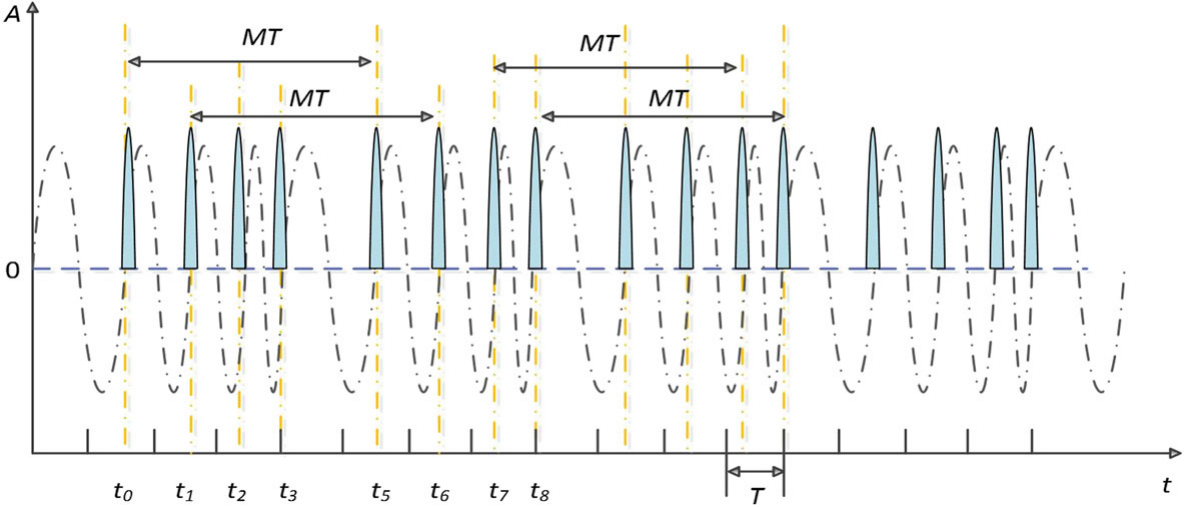
The RF sampling clock function with sinusoid phase modulated and the stream of short pluses

In summary, the stream of short pluses from sinusoid phase modulation function is periodic nonuniform, whose average sampling frequency is *f*_*s*_ = 1/*T* as shown in Fig. 3. Conveniently, choosing *f*_*s*_ = *Mf*_*θ*_ (*M* ∈ *Z*), we can get the period of the short pluses $M T$. Then, the NYFR is periodic nonuniform sampling via the short pluses directly, and there are *M* samples in one period.

## Spectral reconstruction of NYFR

In the abovementioned that the RF sample clock function of NYFR is periodic nonuniform, the RF inputs *s*(*t*_*n*_) are sampled in NYFR that can be represented as

8 $$ s\left({t}_n\right)= s\left({t}_m+ lMT\right) $$

where *m* ∈ {0, 1, 2, …, *M* − 1} is the index of sample time in one period, *l* ∈ {0, 1, …, *ceil*(*N*/*M*) − 1} is the index of period, and ceil(⋅) denotes round up. And *t*_*m*_ = *mT* − *r*_*m*_ *T* as shown in reference [12].

As we know that the sample time *t*_*m*_ can be taken as the phase *ϕ*(*t*) crosses multiple of 2*π*, we have

9 $$ 2\pi {f}_s{t}_m+ \sin \left(2\pi {f}_{\theta}{t}_m\right)=2\pi m $$

and then

10 $$ {t}_m=\frac{2\pi m- \sin \left(2\pi {f}_{\theta}{t}_m\right)}{2\pi {f}_s} $$

Substituting (10) and *t*_*m*_ = *mT* − *r*_*m*_ *T* into the Eq. (6) by reference [13], we have

11 $$ \begin{array}{l}\tilde{A}(l)=\frac{1}{M}{\displaystyle \sum_{m=0}^{M-1}{e}^{- jl{r}_m\frac{2\pi}{M}}{e}^{- jl m\frac{2\pi}{M}}}=\frac{1}{M}{\displaystyle \sum_{m=0}^{M-1}{e}^{- jl2\pi {f}_{\theta}{t}_m}}\\ {}\kern4em =\frac{1}{M}{\displaystyle \sum_{m=0}^{M-1}{e}^{- jl2\pi {f}_{\theta}\left(\frac{2\pi m- \sin \left(2\pi {f}_{\theta}{t}_m\right)}{2\pi {f}_s}\right)}}\\ {}\kern4em ={\displaystyle \sum_{m=0}^{M-1}\left(\frac{1}{M}{e}^{+ j\frac{l}{M} \sin \left(2\pi {f}_{\theta}{t}_m\right)}\right){e}^{- jl\frac{2\pi}{M} m}}\end{array} $$

Eq. (11) means that *Ã*(*l*) is the Fourier transform of the sinusoid modulation function. To simplify *Ã*(*l*), we use Eq. (7) as shown in reference [16]. In the equation, *p*(*t*) is a pulse model, and *k* represents the index of Nyquist zone (NZ) from zero to *κ*, where *κ* denotes the number of NZ by NYFR covered. So *k* can be obtained from *l*, that is to say *k* = ⌊(*l* + *M*/2)/*M*⌋ ∈ *Z* and ⌊ ⋅ ⌋ denotes floor.

As signal modulation theory’s point of view, $2\pi {f}_s{\displaystyle {\sum}_k{e}^{jk\left[2\pi {f}_s t+\theta (t)\right]}}$ can modulate the RF inputs again without convolution with *p*(*t*). So the expression of *Ã*(*l*) can be simplified to

12 $$ \tilde{A}(l)={\displaystyle \sum_{m=0}^{M-1}\left(\frac{1}{M}{e}^{+ jk \sin \left(2\pi {f}_{\theta}{t}_m\right)}\right){e}^{- jl\frac{2\pi}{M} m}} $$

when − *M*/2 + 1 ≤ *l* < *M*/2, the index of NZ is *k* = ⌊(*l* + *M*/2)/*M*⌋ = 0; likewise, when *M*/2 + 1 ≤ *l* < 3*M*/2 corresponds to *k* = ⌊(*l* + *M*/2)/*M*⌋ = 1, et al. And the analysis object turns from a point into a zone.

Using the Jacobi identity

13 $$ \exp \left( j\upsilon \sin \beta \right)={\displaystyle \sum_{\nu =-\infty}^{+\infty }{J}_{\nu}\left(\upsilon \right)} \exp \left( j\nu \beta \right) $$

we can get

14 $$ \tilde{p}(t)=2\pi {f}_s{\displaystyle \sum_{k=0}^{\kappa}{\displaystyle \sum_{\nu =-\infty}^{+\infty }{J}_{\nu}(k)} \exp \left( j2\pi {f}_s kt+ j2\pi {f}_{\theta}\nu t\right)} $$

**Fig. 4:**
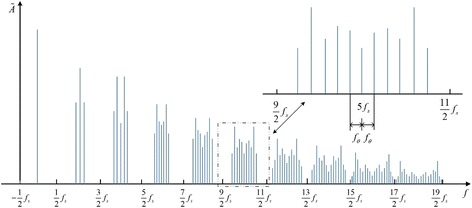
The spectral of a stream of periodic nonuniformly short pluses

Finally, we can obtain some important properties of the spectrum of $\tilde{p}(t)$. The spectrum is centered on the multiple of the average sampling rate *f*_*s*_, and the edge frequencies separated by the amount of *f*_*θ*_. The amplitudes of its edge frequencies satisfy Bessel’s function. Moreover, each NZ of the spectrum comprises *M* lines spaced on the frequency axis *f* uniformly, and the maximum magnitude is related with the index number of NZ as shown in Fig. 4 below.

Based on such a feature, we will extend the spectral reconstruction algorithm of JENQ which is based on time series decomposed model to subsampling. Now, let us consider an arbitrary input frequency *ω*_0_, which limited to ((−1 + 2*k*_*H*_)*πf*_*s*_, (1 + 2*k*_*H*_)*πf*] where *k*_*H*_ = round (*f*_*c*_/*f*_*s*_) is the index of NZ of the input, and the reduction of the summation range which as the Eq. (7) proposed in reference [13] relates to the number of NZ by the band-limited input covered. So the matrix form (8) in the same reference can be changed as

15 $$ \tilde{\mathbf{S}}\left({\omega}_0\right)=\mathbf{A}{\mathbf{S}}_{\mathbf{a}}\left({\omega}_0\right)/ T $$

where the vector $\tilde{\mathbf{S}}\left({\omega}_0\right)$ is the digital spectrum of periodic nonuniformly subsampled signals, and it is expressed as

16 $$ \tilde{\mathbf{S}}\left({\omega}_0\right)={\left[\tilde{S}\left({\omega}_0\right),\tilde{S}\left({\omega}_0+\frac{2\pi}{ M T}\right),\tilde{S}\left({\omega}_0+2\frac{2\pi}{ M T}\right),\dots, \tilde{S}\left({\omega}_0+\left( M-1\right)\frac{2\pi}{ M T}\right)\right]}_{M\times 1}^T $$

The amplitude matrix **A** is

17 $$ \mathbf{A}={\left[\begin{array}{cccc}\hfill A\left(\frac{M}{2}+{k}_H M\right)\hfill & \hfill A\left(\frac{M}{2}+{k}_H M-1\right)\hfill & \hfill \cdots \hfill & \hfill A\left(-\frac{M}{2}+{k}_H M+1\right)\hfill \\ {}\hfill A\left(\frac{M}{2}+{k}_H M+1\right)\hfill & \hfill A\left(\frac{M}{2}+{k}_H M\right)\hfill & \hfill \cdots \hfill & \hfill A\left(-\frac{M}{2}+{k}_H M+2\right)\hfill \\ {}\hfill \vdots \hfill & \hfill \vdots \hfill & \hfill \ddots \hfill & \hfill \vdots \hfill \\ {}\hfill A\left(\frac{M}{2}+{k}_H M+ M-1\right)\hfill & \hfill A\left(\frac{M}{2}+{k}_H M+ M-2\right)\hfill & \hfill \cdots \hfill & \hfill A\left(\frac{M}{2}+{k}_H M\right)\hfill \end{array}\right]}_{M\times M} $$

The vector **S**_**a**_(*ω*_0_) is the Fourier spectrum of the original input signals, and it is expressed as

18 $$ {\mathbf{S}}_{\mathbf{a}}\left({\omega}_0\right)={\left[\begin{array}{c}\hfill {S}_a\left({\omega}_0-\left(\frac{M}{2}+{k}_H M\right)\frac{2\pi}{ M T}\right)\hfill \\ {}\hfill {S}_a\left({\omega}_0-\left(\frac{M}{2}+{k}_H M-1\right)\frac{2\pi}{ M T}\right)\hfill \\ {}\hfill \vdots \hfill \\ {}\hfill {S}_a\left({\omega}_0-\left(-\frac{M}{2}+{k}_H M+1\right)\frac{2\pi}{ M T}\right)\hfill \end{array}\right]}_{M\times 1} $$

Then, the original signal spectrum can be obtained by the following equation:

19 $$ {\mathbf{S}}_{\mathbf{a}}\left({\omega}_0\right)= T{\mathbf{A}}^{-1}\tilde{\mathbf{S}}\left({\omega}_0\right) $$

It is noted that the matrix **A** is column orthogonality, and then, the matrix **A** ^− 1^ exists certainly. However, we need to reevaluate the matrix for each different index value of NZ. Finally, by choosing different value of *ω*_0_, we can get enough uniformly sampled points of the original signal spectrum. And by scanning *k*_*H*_ from zero to *κ*, we can reconstruct the spectrum of NYFR.

## Simulation results and discussion

We take an example for a LFM signal under large bandwidth to show the validity of the proposed method. And the simulation settings are listed in the Table 1.

**Table 1:** The simulation settings table

Average sampling frequency	*f*_*s*_	1 GHz
Sinusoid modulation frequency	*f*_*θ*_	10 MHz
Simulation points	*N*	1000 points
Amplitude of LFM	*A*_0_	1
Initial phase of LFM	*φ*_0_	0
Initial frequency of LFM	*f*_0_	3.52 GHz
Bandwidth of LFM	*B*_0_	0.95 GHz
Modulation rate of LFM	*k*_0_	9.5e6 GHz

**Fig. 5:**
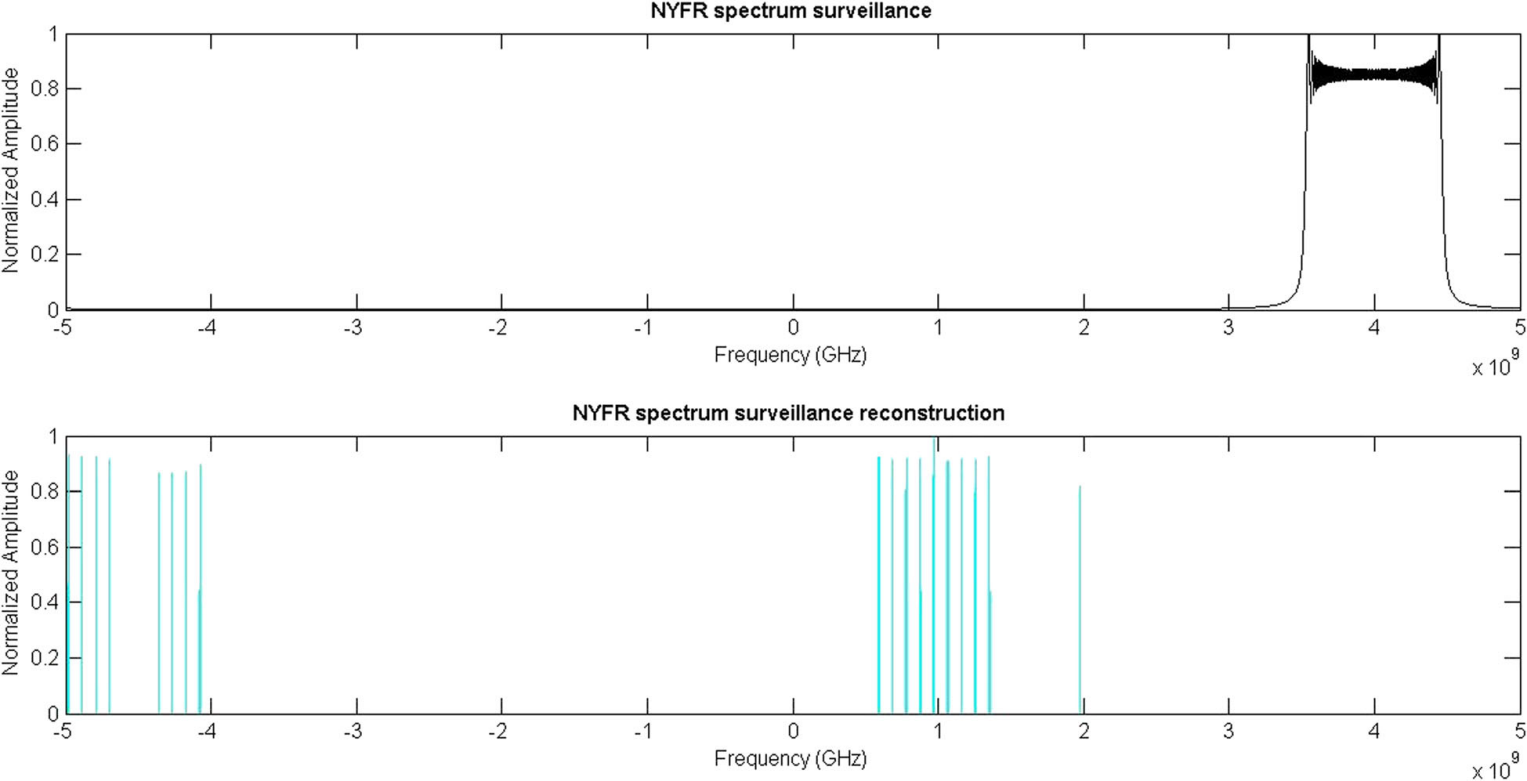
The NYFR spectrum surveillance and its reconstruction

It is assumed that NYFR covers ten Nyquist zones, then the coverage of NYFR spectrum surveillance is from −5GHz to 5GHz. The spectrum of the LFM signal and its OMP reconstruction is shown in Fig. 5. We can see that the signal is not sparse in frequency domain, and the accurate reconstruction cannot be implemented by the existing CS algorithms.

**Fig. 6:**
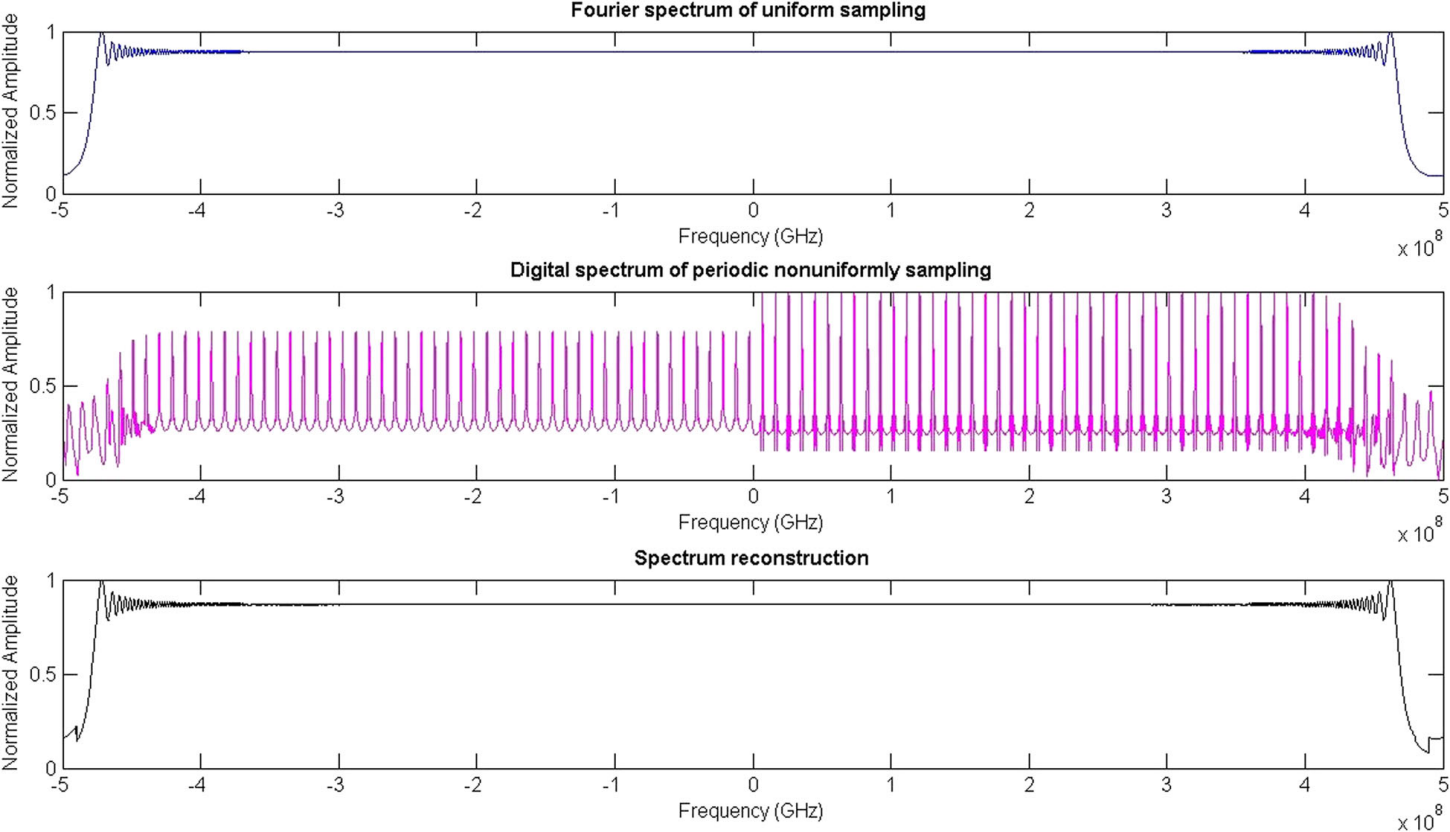
The LFM signal spectrum

In Fig. 6, the Fourier spectrum of the received LFM signal which limited to only one NZ is shown in (a); and for the index of NZ of this signal is *k*_*H*_ = 4, the digital spectrum of periodic nonuniform subsampling is shown in (b); and the figure (c) proved that the proposed spectral reconstruction algorithm is useful. Then, calculating the reconstruction error by 100 times of Monte Carlo experiment is 0.0084, where root-mean-square is used to define the reconstruction error as follows:

20 $$ \varepsilon =\sqrt{\left({\displaystyle \sum_{n=0}^{N-1}{\left|{S}_a\left({\omega}_n\right)-\tilde{S}\left({\omega}_n\right)\right|}^2}\right)/ N} $$

- Fourier spectrum of uniform sampling
- Digital spectrum of periodic nonuniform subsampling
- Spectral reconstruction

## Conclusions

NYFR is an efficient A2I conversion model, and its spectral reconstruction can use the traditional CS recovery algorithms. However, if the signal is not sparse in frequency domain as shown in simulation, the existing CS algorithms as OMP cannot reconstruct the received signal accurately. In this paper, we first derive that the RF sample clock function of NYFR is periodic nonuniform. Then, the classical results of periodic nonuniform sampling are applied to NYFR. We extend the spectral reconstruction algorithm of time series decomposed model to the subsampling case by using the spectrum characteristics of NYFR. And finally, we take an example for a LFM signal under large bandwidth to verify the proposed algorithm and compare the spectral reconstruction algorithm with OMP algorithm. But for the influence of noise, the parameter estimation of wideband LFM signals will be more difficult. In the future work, we will study the fractional spectrum reconstruction of periodic nonuniform subsampling and their applications.

## Abbreviations

A2I: Analogy-to-Information
ADC: Analog to digital converter
CS: Compressive sensing
LCM: Least common multiple
LFM: Linear frequency modulation
NYFR: Nyquist folding receiver
NZ: Nyquist zone
OMP: Orthogonal matching pursuit
RF: Radio frequency
RIP: Restricted isometry property
SFM: Sinusoid frequency modulation
